# Lysozyme Gene Expression in 3T3-L1 Cells Sustains Expression of Adipogenic Genes and Adipocyte Differentiation

**DOI:** 10.3389/fcell.2022.914788

**Published:** 2022-06-15

**Authors:** Aina Lluch, Jessica Latorre, José Manuel Fernández-Real, José María Moreno-Navarrete

**Affiliations:** ^1^ Department of Diabetes, Endocrinology and Nutrition, Institut D’Investigació Biomèdica de Girona (IdIBGi), Girona, Spain; ^2^ CIBER Fisiopatología de la Obesidad y Nutrición (CIBERobn, CB06/ 03/010), Instituto de Salud Carlos III, Girona, Spain; ^3^ Department of Medicine, Universitat de Girona, Girona, Spain

**Keywords:** lysozyme, 3T3-L1, adipogenesis, gene knockdown, lentiviral particles

## Abstract

Substantial levels of lysozyme in adipose tissue in association to obesity have been recently demonstrated in mice and humans. In addition, experiments in mice suggest that lysozyme might impact on adipose tissue adipogenesis. To further investigate the relationship between lysozyme and adipogenesis, in the present study, we aimed to study lysozyme (Lyz2) during 3T3-L1 adipocyte differentiation and its possible role in adipogenesis. Time course experiment during 3T3-L1 adipocyte differentiation indicated that *Lyz2* gene expression decreased at day 4, which was caused by isobutylmethylxanthine administration, and recovered at the end of the process (day 8). Importantly, the impact of isobutylmethylxanthine-induced downregulation of *Lyz2* gene expression on adipogenesis was not comparable to that observed in the full cocktail, questioning whether the reduction in lysozyme at early stage of adipocyte differentiation is relevant to this process. In fact, the depletion in *Lyz2* expression had a negative impact on adipogenesis, and rosiglitazone administration failed to compensate for the anti-adipogenic effect observed in *Lyz2* gene knockdown cells. Otherwise, when *Lyz2* gene knockdown cells were co-cultured with control cells, these cells had higher expression of adipogenic genes than those co-cultured with themselves at the end of adipocyte differentiation. In conclusion, this study suggests that lysozyme expression in 3T3-L1 cells sustains expression of adipogenic genes and adipocyte differentiation.

## Introduction

The generation of new adipocytes in adipose tissue is mediated by adipogenesis, which consists in the differentiation of adipose-derived mesenchymal stem cells into adipocytes. This process is crucial to maintain adipose tissue physiology and prevent obesity-associated metabolic disturbances (Vishvanath and Gupta, 2019).

In the searching of new factors that might modulate adipose tissue adipogenesis, in a recent study, we found that lysozyme was highly expressed in adipose tissue in association with obesity and inflammation, but negatively correlated with the expression of adipogenesis-related genes in both subcutaneous and visceral adipose tissue (Latorre et al., 2021). More importantly, adipose tissue lysozyme gene knockdown in mice fed with high-fat and high-sucrose diet improved adipogenesis in parallel to reduced inflammatory markers (Latorre et al., 2021).

Although the highest expression of lysozyme in adipose tissue occurs in macrophages, it is also expressed to a lesser extent in preadipocytes and adipocytes (Latorre et al., 2021). At cellular level, lysozyme expression during adipogenesis, and whether it has a direct role on this process remains to be explored. In the present study, we aimed to investigate lysozyme (Lyz2) during 3T3-L1 adipocyte differentiation and its possible role in this process.

## Materials and Methods

### Cell Culture and Differentiation

3T3-L1 cells, an embryonic fibroblast mouse cell line (ATCC, LGC Standards GmbH, Wesel, Germany), were cultured in Dulbecco’s Modified Eagle’s Medium (DMEM) containing 4.5 g/L glucose supplemented with 10% Fetal Bovine Serum (FBS), 1% penicillin and streptomycin, 1% Glutamine and 2% Hepes at 37°C and 5% CO_2_. At 2 days after confluence, to induce differentiation, a cocktail mixture containing insulin (INS, 5 μg/ml), dexamethasone (DEX, 0.25 μM), and isobutylmethylxanthine (IBMX, 0.25 mM) was added for 4 days (Day 4), followed by 4 days with INS (5 μg/ml) alone (Day 8). Cocktail components were tested separately at Day 4, comprising INS (5 μg/ml) or DEX (0.25 μM) or IBMX (0.25 mM) or all of them.

### *Lyz2* Silencing by Lentiviral Particles

Permanent silencing was achieved in murine 3T3-L1 fibroblasts using *Lyz2*-targeted and control sh-RNA lentiviral particles, obtained as previously described (Latorre et al., 2021). Puromycin (2 ug/ml) was used to select positive adipocytes harboring sh-RNA cassette of *Lyz2* or control. After selection, cells were induced to differentiate, comprising Day 0, Day 4 and Day 8. Afterwards, Rosiglitazone treatments at 0.5 μmol/L were applied in shRNA_SCR and shRNA_Lyz2 cells in parallel to vehicle-treated shRNA_SCR and shRNA_Lyz2 cells, all induced to differentiate into mature adipocytes.

### Coculture of *Lyz2* Silenced and Control Cells

Coculture was assessed using cell culture inserts 0.4 um pore size (#353180, Corning Inc., Corning, NY) in a TC-Platte 12 Well (#83.3921, Sarstedt, Nümbrecht, Germany) making the combinations of shRNA_Lyz2 (upper) and shRNA_Lyz2 or shRNA_SCR (down) and induced to differentiate into mature adipocytes.

### Gene Expression

Total RNA was extracted from cells using RNeasy Mini Kit (QIAgen, Izasa SA, Barcelona, Spain) and quantified using a Nanodrop ND-1000 Spectrophotometer (Thermo Fisher Scientific, Waltham, MA, United States). The same amount of total RNA was reverse transcribed to cDNA using High Capacity cDNA Archive Kit (Applied Biosystems, Darmstadt, Germany). Commercial available TaqMan primer/probes sets were used to analyze gene expression with a Light Cycler 480 II (Roche Diagnostics SL, Barcelona, Spain). Primer/probe sets used were as follows: Peptidylprolyl isomerase A (*Ppia*, Mm02342430_g1 as endogenous control), lysozyme (*Lyz2*, Mm01612741_m1), CCAAT/enhancer binding protein (C/EBP), alpha (*Cebpa*, Mm00514283_s1), CCAAT/enhancer binding protein (C/EBP), beta (*Cebpb*, Mm00843434_s1), fatty acid binding protein 4, adipocyte (*Fabp4*, Mm00445880_m1), adiponectin (*Adipoq*, Mm04933656_m1), perilipin 1 (*Plin1*, Mm00558672_m1), solute carrier family 2 member 4 (*Slc2a4*, Mm00436615_m1) and peroxisome proliferator activated receptor gamma (*Pparg*, Mm00440940_m1).

### Oil-Red O Staining

Oil-Red O Staining was used to assess intracellular lipid content. Briefly, cells were washed with cold PBS, fixed with paraformaldehyde 4% (Sigma-Aldrich, Saint Louis, MO, United States) for 1 h, dipped in isopropanol 60% before completely dried, stained with filtered 5% Oil-Red O solution in isopropanol for 10 min at room temperature and washed several times with distilled water. Images were taken using an inverted microscope Anxiovert 40 CFL (Carl Zeiss, Jena, Germany). The stained area was quantified with ImageJ software (Rasband, W. S., ImageJ, United States National Institutes of Health, Bethesda, MD) by means of optimal applicable threshold tools. The image was first transformed to 8-bit format and inverted, followed by a constant threshold adjustment for all conditions, allowing to distinct the red-oil stained areas from the rest with red signal, which was analyzed as the % stained area in respect to image total area.

### Statistical Analysis

Statistical analyses were performed using SPSS statistical software (SPSS v21.0; IBM, Chicago, IL, United States). Data are presented as mean ± SEM. Unpaired *t*-test and nonparametric test (Mann Whitney test) was used to analyse *in vitro* experimental data. Levels of statistical significance were set at *p* < 0.05.

## Results

### *Lyz2* Gene Expression During 3T3-L1 Adipocyte Differentiation

**FIGURE 1:**
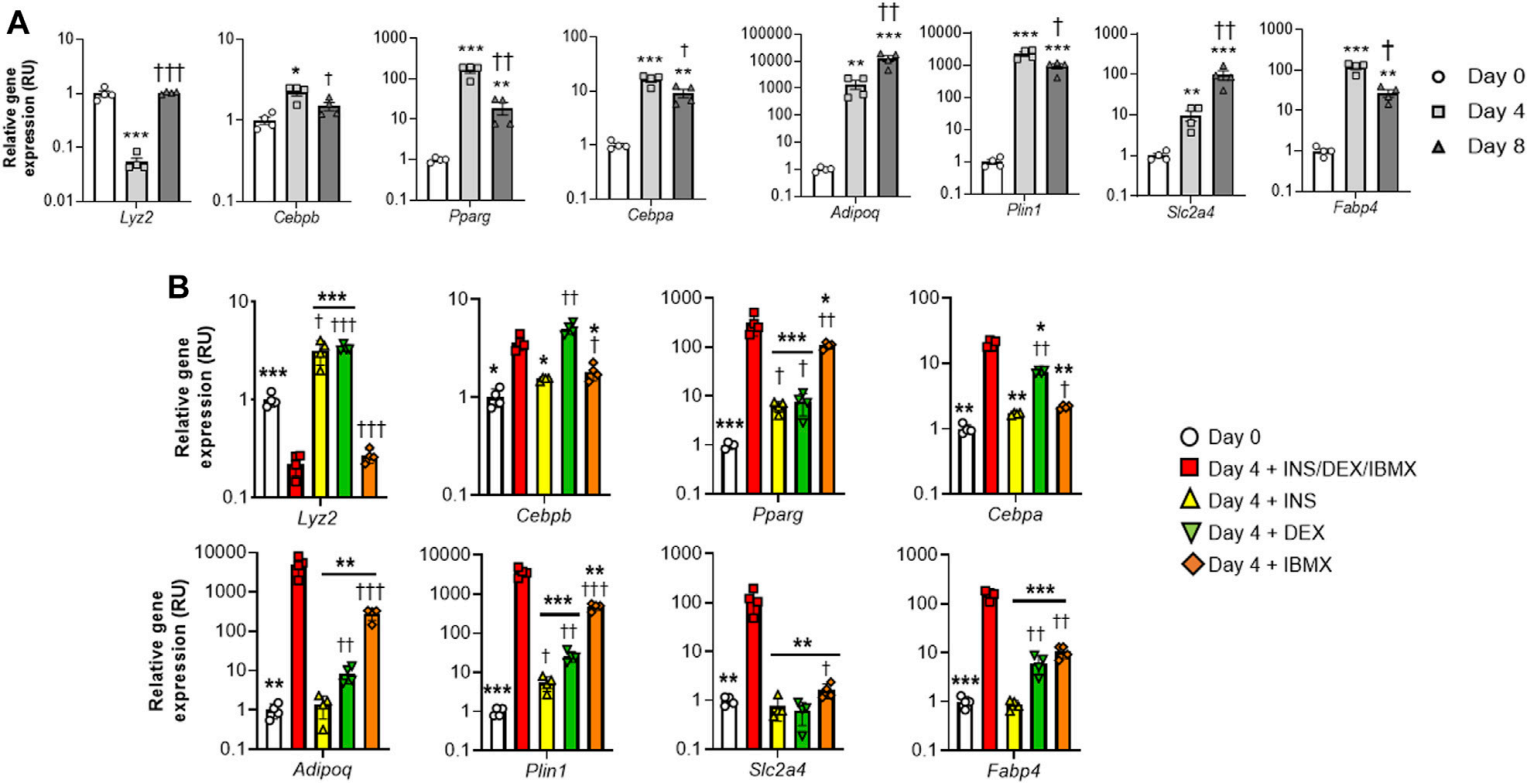
*Lyz2* gene expression during 3T3-L1 adipocyte differentiation. **(A)** *Lyz2, Cebpb, Pparg, Cebpa, Adipoq, Plin1, Slc2a4* and *Fabp4* gene expression during 3T3-L1 adipocyte differentiation. **p < 0.01 and ***p < 0.001 compared to Day 0; ^†^ *p* < 0.05, ^††^ *p* < 0.01 and ^†††^ *p* < 0.001 compared to Day 4. **(B)** The effect of individual compound administration (INS, DEX or IBMX) of differentiation cocktail on *Lyz2, Cebpb, Pparg, Cebpa, Adipoq, Plin1, Slc2a4* and *Fabp4* gene expression during 4 days. **p < 0.01 and ***p < 0.001 compared to complete differentiation cocktail (INS/DEX/IBMX); ^†^ *p* < 0.05, ^††^ *p* < 0.01 and ^†††^ *p* < 0.001 compared to Day 0.

In a time course experiment, we found that *Lyz2* gene expression decreased at day 4, but was recovered at the end of the process (day 8) (Figure 1A). Interestingly, the decrease of *Lyz2* gene expression in this early stage of adipocyte differentiation was associated with the increase in gene expression of very early (*Cebpb*), early (*Cebpa, Pparg*) and middle-terminal stage (*Adipoq, Plin1, Slc2a4* and *Fabp4*) markers of adipogenesis (Farmer, 2006; Kleiman et al., 2009; Burrell and Stephens, 2021; Yadav and Jang, 2021) (Figure 1A), suggesting that the decrease in *Lyz2* gene expression at this stage might be required for adipocyte differentiation.

To study which compound of the differentiation cocktail could be responsible for the downregulation of *Lyz2* gene expression at day 4, a second experiment in which individual administrations of insulin, dexamethasone or isobutylmethylxanthine (IBMX) was performed, only IBMX replicated the observed effects of the complete differentiation cocktail (Figure 1B). In fact, insulin and dexamethasone administrations displayed opposite effects (Figure 1B). In this experiment, even though insulin- and dexamethasone-induced increase of *Lyz2* gene expression was associated to decreased expression in adipogenic genes compared to the full cocktail administration, the impact of IBMX-induced downregulation of *Lyz2* gene expression on adipogenesis was not comparable to that observed in the full cocktail (Figure 1B), questioning whether the reduction in lysozyme is relevant to adipocyte differentiation.

To address this point, permanent *Lyz2* gene knockdown (KD) was performed and assessed during adipocyte differentiation.

### Knockdown of *Lyz2* Gene Inhibits Adipogenic Gene Expression

**FIGURE 2:**
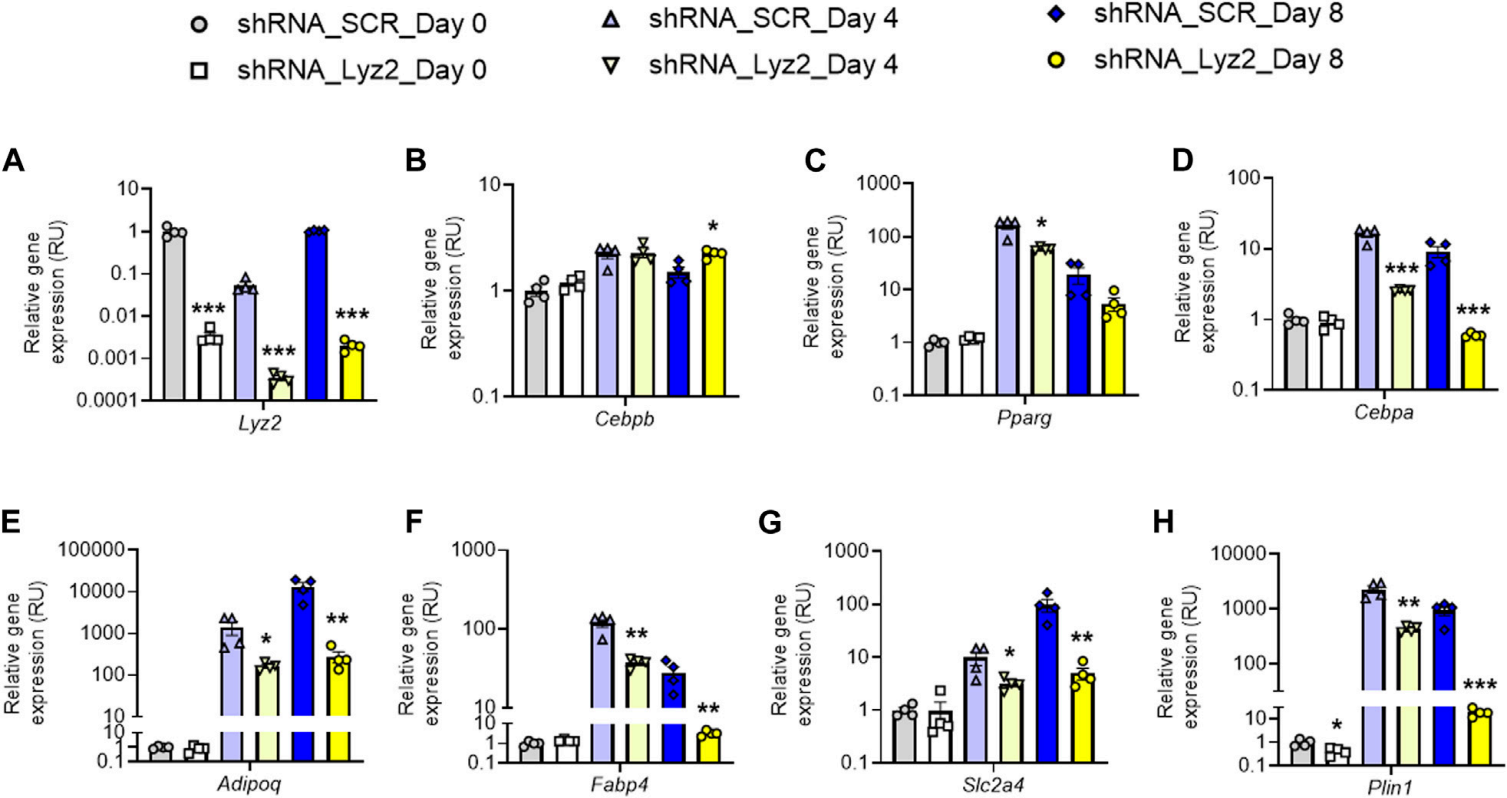
The impact of *Lyz2* gene knockdown on expression of adipogenic genes during 3T3-L1 adipocyte differentiation. **(A–H)** *Lyz2, Cebpb, Pparg, Cebpa, Adipoq, Plin1, Slc2a4* and *Fabp4* gene expression during 3T3-L1 adipocyte differentiation at day 0, 4 and 8. *p < 0.05, **p < 0.01 and ***p < 0.001 compared to shRNA_SCR.

**FIGURE 3:**
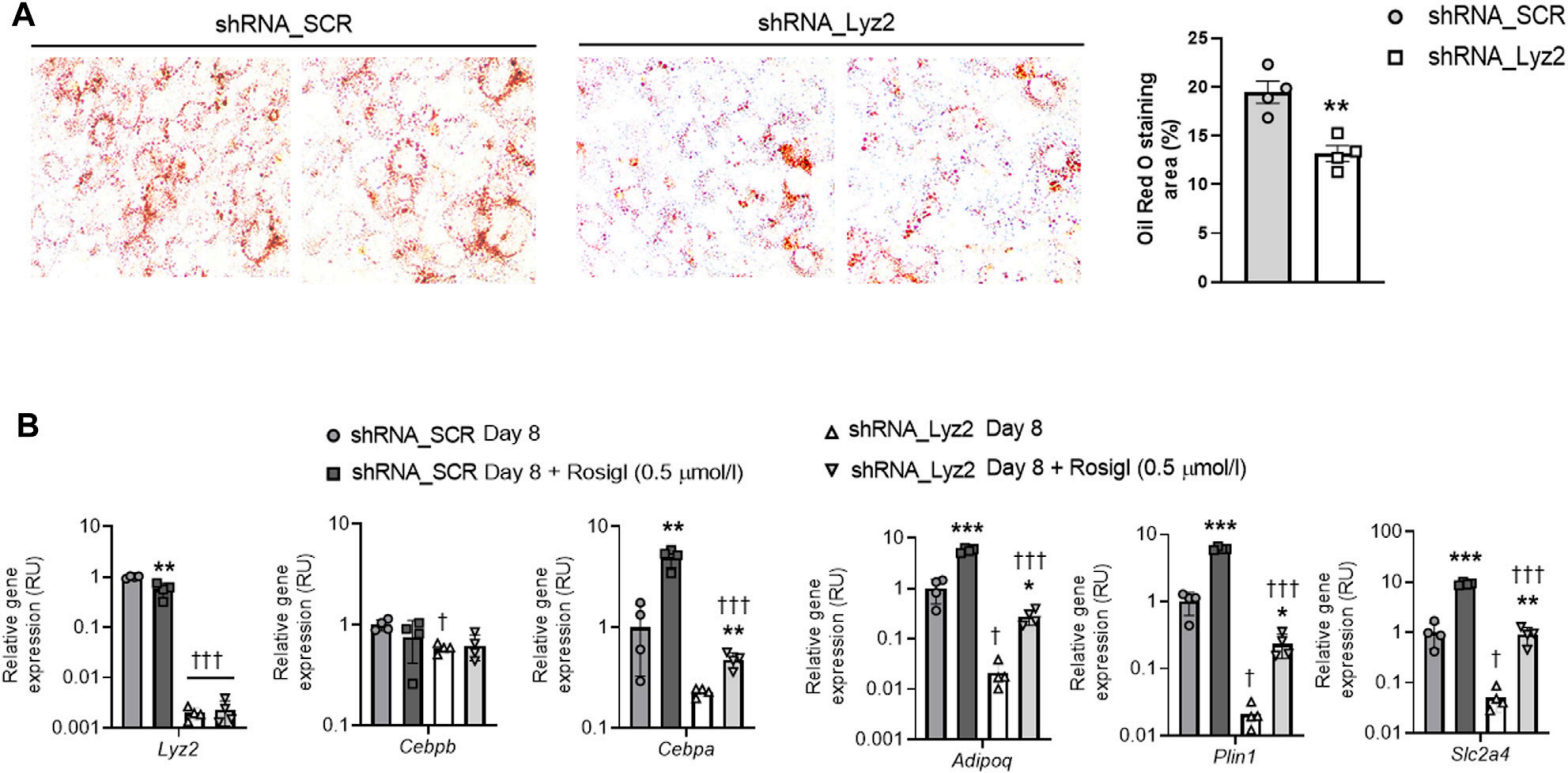
**(A)** The impact of *Lyz2* gene knockdown on intracellular lipid accumulation, which was measured as Oil Red O staining area (%), at day 8 of adipocyte differentiation. The lens magnification used was ×10. **p < 0.01 compared to shRNA_SCR. **(B)** The impact of rosiglitazone (0.5 μmol/L) administration during adipocyte differentiation on *Lyz2, Cebpb, Cebpa, Adipoq, Plin1* and *Slc2a4* gene expression at day 8 in shRNA_SCR and shRNA_Lyz2 cells. *p < 0.05, **p < 0.01 and ***p < 0.001 compared to vehicle; ^†^ *p* < 0.05 and ^†††^ *p* < 0.001 compared to shRNA_SCR.

At day 0, *Lyz2* gene KD slightly decreased *Plin1* gene expression, without significant changes in the other adipogenic gene expression (Figures 2A−H). At day 4, *Lyz2* gene KD reduced expression of early (*Cebpa, Pparg*) and middle-terminal stage (*Adipoq, Plin1, Slc2a4* and *Fabp4*) of adipogenesis-related genes, and finally this attenuation was enhanced on *Cebpa*, *Adipoq, Plin1, Slc2a4* and *Fabp4* gene expression at day 8 (Figures 2A−H), indicating that the depletion in *Lyz2* expression had a negative impact on adipogenesis. In line with this, intracellular lipid accumulation measured by Oil Red O staining at day 8 was significantly reduced in *Lyz2* gene KD cells (Figure 3A).

### The Anti-Adipogenic Effect of *Lyz2* Gene KD Is Not Overcome by Rosiglitazone

In control cells, rosiglitazone administration (0.5 μmol/L) led to decreased *Lyz2* gene expression, and as expected, in both control and *Lyz2* gene KD cells enhanced expression of adipogenic genes (Figure 3B). However, rosiglitazone administration (0.5 μmol/L) failed to compensate for the anti-adipogenic effect observed in *Lyz2* gene KD compared rosiglitazone-treated control cells (Figure 3B).

### Co-Culture of *Lyz2* Gene KD With Control Cells Improves Adipogenic Gene Expression

**FIGURE 4:**
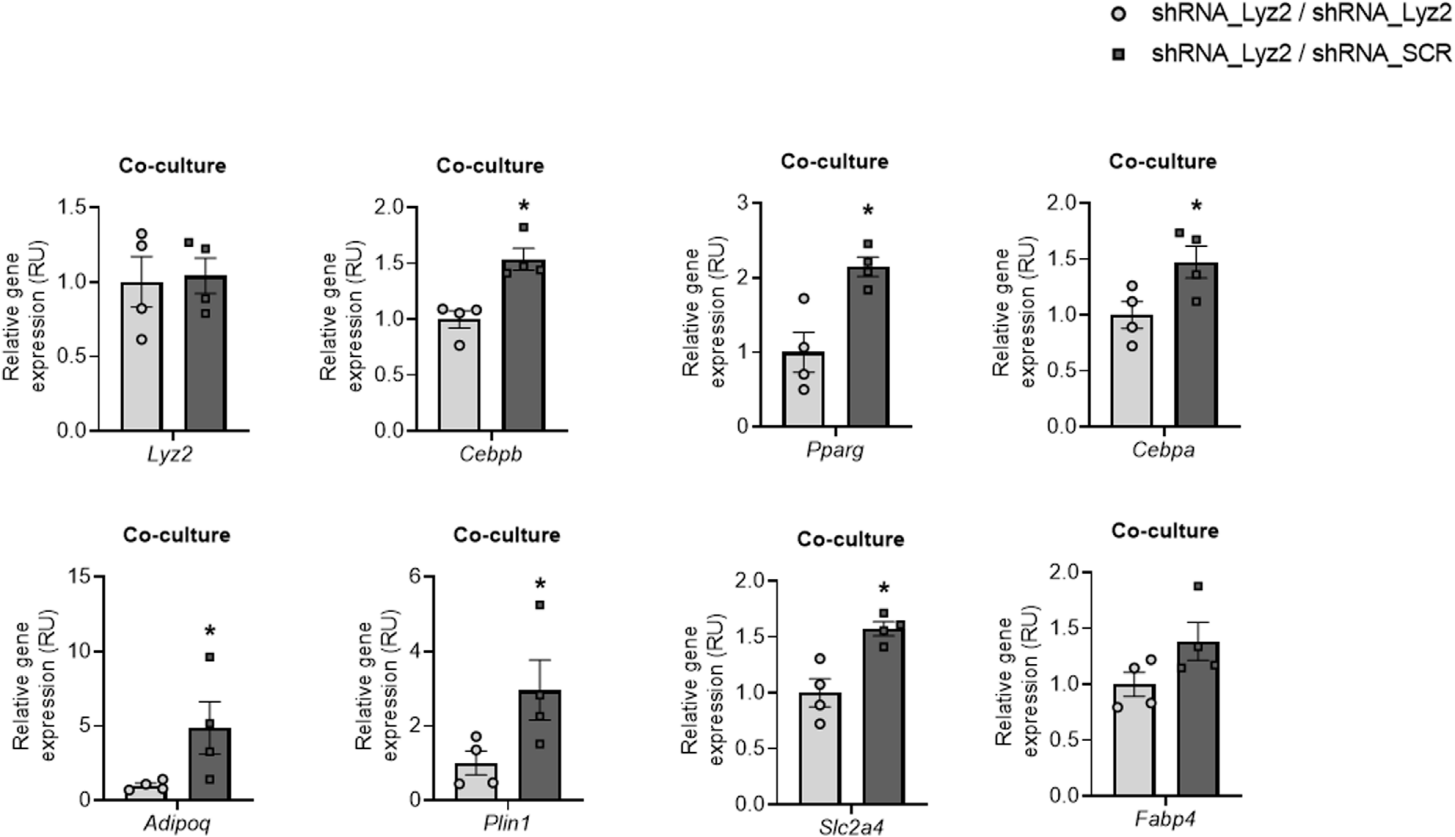
The effect of coculture *Lyz2* gene knockdown cells (shRNA_Lyz2) with lysozyme-producing cells (shRNA_SCR) on *Lyz2, Cebpb, Pparg, Cebpa, Adipoq, Plin1, Slc2a4* and *Fabp4* gene expression at day 8 of adipocyte differentiation. *p < 0.05 compared to shRNA_Lyz2 cells co-cultured with themselves.

Then, the effects of lysozyme biosynthesis from control cells on *Lyz2* gene KD cells were assessed in a co-culture experiment using transwell insert microporous membranes, as previously reported (Moreno-Navarrete et al., 2015). Of note, *Lyz2* gene KD cells co-cultured with control cells had higher expression of adipogenic genes than those co-cultured with themselves (Figure 4).

## Discussion

To the best of our knowledge this is the first study evaluating the direct role of preadipocyte- and adipocyte-produced lysozyme on adipogenesis. Altogether, these data indicate that *Lyz2* gene knockdown (greater than 95%) resulted in a significant inhibition in 3T3-L1 adipocyte differentiation, characterized by reduced expression of adipogenic genes and decreased intracellular lipid accumulation. Compared to day 4, *Plin1* and *Fabp4* mRNA decreased at day 8. This might indicate that adipocyte maturation was completely achieved at day8. Even though previous studies demonstrated that gene expression of these adipogenic genes ran in parallel to their protein levels (Kleiman et al., 2009; Burrell and Stephens, 2021; Yadav and Jang, 2021), the impact of *Lyz2* gene KD on these adipogenic genes at protein level should be confirmed in future studies.

Supporting the impact of lysozyme on fat mass expansion, studies in breastfeeding infants reported that calculated daily intake of lysozyme was positively associated to infant fat mass (Gridneva et al., 2021; Gridneva et al., 2022), and studies in humans, mice and rats demonstrated increased adipose tissue lysozyme in association to adiposity (Latorre et al., 2021) or increased plasma lysozyme in association to fat mass (Moreno-Navarrete et al., 2021). However, in contrast to the importance of lysozyme to maintain adipogenesis in 3T3-L1 cells, lysozyme expression in human and mice adipose tissue was negatively associated to gene expression markers of adipogenesis, but positively with adipose tissue inflammatory markers (Latorre et al., 2021). Taking into account that macrophages were the main source of lysozyme in adipose tissue (Latorre et al., 2021), these discrepancies might indicate that adipose tissue lysozyme reflects the proinflammatory effect of macrophage lysozyme (Shimada et al., 2010; Davis et al., 2011), and the consequent negative effect of inflammation on adipogenesis (Vila et al., 2014; Kursawe et al., 2016). In fact, experimentally, the downregulation of adipose tissue lysozyme impacted on adipose tissue, attenuating local adipose tissue inflammatory activity and improving adipogenesis (Latorre et al., 2021).

The beneficial effects of lysozyme on adipocyte biology could be explained in part by its protective role against the deleterious effects of advanced glycation end-products (AGEs) (Mitsuhashi et al., 1997; Cocchietto et al., 2008; Gallo et al., 2014). However, the absence of AGEs in current experiments suggest other mechanisms. Previous studies demonstrated that lysozyme can exert antioxidant activities through the suppression of endogenous reactive oxygen species (ROS) (Liu et al., 2006a; Liu et al., 2006b) and attenuating the ROS harmful effects in hepatocytes (Liu et al., 2006a). Taking into account that intracellular ROS levels increased during 3T3-L1 adipocyte differentiation, and that excess intracellular ROS impaired expression of adipogenic genes and resulted in a pathological adipocyte differentiation (Furukawa et al., 2004), a possible role of lysozyme buffering the negative impact of endogenous ROS on adipocyte differentiation might be postulated. However, further experiments should be designed to investigate the molecular mechanism that underlie in the requirement of lysozyme expression for an optimal adipocyte differentiation.

This study also reported different effects in the regulation of *Lyz2* gene expression of the components that constitute the adipocyte differentiation cocktail. Of note, insulin administration led to increased *Lyz2* gene expression in 3T3-L1 cells. These data suggest that obesity-associated hyperinsulinemia could contribute to increased circulating and adipose tissue lysozyme levels observed in subjects with obesity (Latorre et al., 2021; Moreno-Navarrete et al., 2021). Strikingly, dexamethasone administration also increased *Lyz2* gene expression in 3T3-L1 cells. In contrast, dexamethasone administration impacted negatively on lysozyme expression in human monocytic cell lines, such as THP-1 and U937) (Hoff et al., 1992; Kulkarni et al., 2016). Otherwise, and in agreement with current findings, the phosphodiesterase inhibitor (isobutylmethylxanthine) also decreased lysozyme production in neutrophils and monocytes through the increase in cAMP levels (Herlin and Kragballe, 1982; Nagata et al., 1992).

An important limitation of co-culture experiment is that the chambers allow the flow of the entire secretome of control cells, not directly showing if lysozyme alone can rescue the defect. Further experiments treating *Lyz2* gene KD cells with lysozyme should be performed to confirm if this is sufficient to improve adipogenesis in the absence of endogenous lysozyme production. To gain insight in the mechanism behind the observed Lyz2-associated changes in adipogenesis, additional experiments are required. For instance, the bioenergetic profiles of these cells might be evaluated.

In conclusion, these data indicate that lysozyme is required to achieve an optimal adipocyte differentiation in 3T3-L1 cells, suggesting a possible role of lysozyme in the maintenance of adequate adipogenic gene expression.

## Data Availability

The original contributions presented in the study are included in the article, further inquiries can be directed to the corresponding author.

## References

[B1] Burrell J. A. Stephens J. M. (2021). KAT8, Lysine Acetyltransferase 8, Is Required for Adipocyte Differentiation *In Vitro* . Biochim. Biophys. Acta (BBA) - Mol. Basis Dis. 1867 (6), 166103. 10.1016/j.bbadis.2021.166103 PMC8026702 33617987

[B2] Cocchietto M. Zorzin L. Toffoli B. Candido R. Fabris B. Stebel M. (2008). Orally Administered Microencapsulated Lysozyme Downregulates Serum AGE and Reduces the Severity of Early-Stage Diabetic Nephropathy. Diabetes Metab. 34 (6 Pt 1), 587–594. 10.1016/j.diabet.2008.05.009 18926757

[B3] Davis K. M. Nakamura S. Weiser J. N. (2011). Nod2 Sensing of Lysozyme-Digested Peptidoglycan Promotes Macrophage Recruitment and Clearance of S. Pneumoniae Colonization in Mice. J. Clin. Invest. 121 (9), 3666–3676. 10.1172/JCI57761 21841315 PMC3163965

[B4] Farmer S. R. (2006). Transcriptional Control of Adipocyte Formation. Cell Metab. 4 (4), 263–273. 10.1016/j.cmet.2006.07.001 17011499 PMC1958996

[B5] Furukawa S. Fujita T. Shimabukuro M. Iwaki M. Yamada Y. Nakajima Y. (2004). Increased Oxidative Stress in Obesity and its Impact on Metabolic Syndrome. J. Clin. Invest. 114 (12), 1752–1761. 10.1172/JCI21625 15599400 PMC535065

[B6] Gallo D. Cocchietto M. Masat E. Agostinis C. Harei E. Veronesi P. (2014). Human Recombinant Lysozyme Downregulates Advanced Glycation Endproduct-Induced Interleukin-6 Production and Release in an *In-Vitro* Model of Human Proximal Tubular Epithelial Cells. Exp. Biol. Med. (Maywood) 239 (3), 337–346. 10.1177/1535370213518281 24495950

[B7] Gridneva Z. Lai C. T. Rea A. Tie W. J. Ward L. C. Murray K. (2021). Human Milk Immunomodulatory Proteins are Related to Development of Infant Body Composition during the First Year of Lactation. Pediatr. Res. 89 (4), 911–921. 10.1038/s41390-020-0961-z 32438370

[B8] Gridneva Z. Rea A. Lai C. T. Tie W. J. Kugananthan S. Warden A. H. (2022). Human Milk Macronutrients and Bioactive Molecules and Development of Regional Fat Depots in Western Australian Infants during the First 12 Months of Lactation. Life 12 (4), 493. 10.3390/life12040493 35454985 PMC9029383

[B9] Herlin T. Kragballe K. (1982). Divergent Effects of Methylxanthines and Adenylate Cyclase Agonists on Monocyte Cytotoxicity and Cyclic AMP Levels. Eur. J. Clin. Invest. 12 (4), 293–299. 10.1111/j.1365-2362.1982.tb02235.x 6183123

[B10] Hoff T. Spencker T. Emmendoerffer A. Goppelt-Struebe M. (1992). Effects of Glucocorticoids on the TPA-Induced Monocytic Differentiation. J. Leukoc. Biol. 52 (2), 173–182. 10.1002/jlb.52.2.173 1506773

[B11] Kleiman E. Carter G. Ghansah T. Patel N. A. Cooper D. R. (2009). Developmentally Spliced PKCβII Provides a Possible Link between mTORC2 and Akt Kinase to Regulate 3T3-L1 Adipocyte Insulin-Stimulated Glucose Transport. Biochem. Biophys. Res. Commun. 388 (3), 554–559. 10.1016/j.bbrc.2009.08.063 19686698 PMC3033743

[B12] Kulkarni N. N. Gunnarsson H. I. Yi Z. Gudmundsdottir S. Sigurjonsson O. E. Agerberth B. (2016). Glucocorticoid Dexamethasone Down-Regulates Basal and Vitamin D3 Induced Cathelicidin Expression in Human Monocytes and Bronchial Epithelial Cell Line. Immunobiology 221 (2), 245–252. 10.1016/j.imbio.2015.09.001 26358366

[B13] Kursawe R. Dixit V. D. Scherer P. E. Santoro N. Narayan D. Gordillo R. (2016). A Role of the Inflammasome in the Low Storage Capacity of the Abdominal Subcutaneous Adipose Tissue in Obese Adolescents. Diabetes 65 (3), 610–618. 10.2337/db15-1478 26718495 PMC4764142

[B14] Latorre J. Lluch A. Ortega F. J. Gavaldà-Navarro A. Comas F. Morón-Ros S. (2021). Adipose Tissue Knockdown of Lysozyme Reduces Local Inflammation and Improves Adipogenesis in High-Fat Diet-Fed Mice. Pharmacol. Res. 166, 105486. 10.1016/j.phrs.2021.105486 33556481

[B15] Liu H. Zheng F. Cao Q. Ren B. Zhu L. Striker G. (2006a). Amelioration of Oxidant Stress by the Defensin Lysozyme. Am. J. Physiol.-Endocrinol. Metab. 290 (5), E824–E832. 10.1152/ajpendo.00349.2005 16317028

[B16] Liu H. Zheng F. Li Z. Uribarri J. Ren B. Hutter R. (2006b). Reduced Acute Vascular Injury and Atherosclerosis in Hyperlipidemic Mice Transgenic for Lysozyme. Am. J. Pathol. 169 (1), 303–313. 10.2353/ajpath.2006.050885 16816382 PMC1698766

[B17] Mitsuhashi T. Li Y. M. Fishbane S. Vlassara H. (1997). Depletion of Reactive Advanced Glycation Endproducts from Diabetic Uremic Sera Using a Lysozyme-Linked Matrix. J. Clin. Invest. 100 (4), 847–854. 10.1172/JCI119600 9259584 PMC508257

[B18] Moreno-Navarrete J. M. Escoté X. Ortega F. Camps M. Ricart W. Zorzano A. (2015). Lipopolysaccharide Binding Protein Is an Adipokine Involved in the Resilience of the Mouse Adipocyte to Inflammation. Diabetologia 58 (10), 2424–2434. 10.1007/s00125-015-3692-7 26201685

[B19] Moreno-Navarrete J. M. Latorre J. Lluch A. Ortega F. J. Comas F. Arnoriaga-Rodríguez M. (2021). Lysozyme is a Component of the Innate Immune System Linked to Obesity Associated-Chronic Low-Grade Inflammation and Altered Glucose Tolerance. Clin. Nutr. 40 (3), 1420–1429. 10.1016/j.clnu.2020.08.036 32943240

[B20] Nagata S. Kebo D. K. Kunkel S. Glovsky M. (1992). Effect of Adenylate Cyclase Activators on C5a-Lnduced Human Neutrophil Aggregation, Enzyme Release and Superoxide Production. Int. Arch. Allergy Immunol. 97 (3), 194–199. 10.1159/000236118 1375201

[B21] Shimada T. Park B. G. Wolf A. J. Brikos C. Goodridge H. S. Becker C. A. (2010). *Staphylococcus A* Evades Lysozyme-Based Peptidoglycan Digestion that Links Phagocytosis, Inflammasome Activation, and IL-1β Secretion. Cell Host Microbe 7 (1), 38–49. 10.1016/j.chom.2009.12.008 20114027 PMC2818986

[B22] Vila I. K. Badin P.-M. Marques M.-A. Monbrun L. Lefort C. Mir L. (2014). Immune Cell Toll-Like Receptor 4 Mediates the Development of Obesity- and Endotoxemia-Associated Adipose Tissue Fibrosis. Cell Rep. 7 (4), 1116–1129. 10.1016/j.celrep.2014.03.062 24794440

[B23] Vishvanath L. Gupta R. K. (2019). Contribution of Adipogenesis to Healthy Adipose Tissue Expansion in Obesity. J. Clin. Invest. 129 (10), 4022–4031. 10.1172/JCI129191 31573549 PMC6763245

[B24] Yadav A. K. Jang B.-C. (2021). Inhibition of Lipid Accumulation and Cyclooxygenase-2 Expression in Differentiating 3T3-L1 Preadipocytes by Pazopanib, a Multikinase Inhibitor. Int. J. Mol. Sci. 22 (9), 4884. 10.3390/ijms22094884 34063048 PMC8125232

